# Evaluating the Performance of Machine Learning Models for Predicting 5-Year Breast Cancer Survival: A Systematic Review and Meta-Analysis

**DOI:** 10.3390/cancers18172736

**Published:** 2026-08-23

**Authors:** Ashin Krishna Chalil, Bindu Therayangalath, Vikram Patil, Chaithra Nagaraju

**Affiliations:** 1 Division of Medical Statistics, School of Life Sciences, JSS Academy of Higher Education and Research, Mysuru 570015, India; 24plm050@jssuni.edu.in; 2 Department of Cancer Registry & Epidemiology, Malabar Cancer Centre, Postgraduate Institute of Oncology Science & Research, Kannur 670103, India; bindut@mcc.kerala.gov.in; 3 Department of Radio Diagnosis, JSS Medical College & Hospital, JSS Academy of Higher Education and Research, Mysuru 570015, India; vikrampatil@jssuni.edu.in

**Keywords:** breast cancer, machine learning models, 5-year survival prediction, meta-analysis

## Abstract

Background: Breast cancer is the most common malignancy among women worldwide and a leading cause of cancer-related mortality. Despite advances in diagnosis and treatment, accurately predicting 5-year survival remains challenging because of disease heterogeneity. This study systematically evaluated the performance of machine learning (ML) models for predicting 5-year breast cancer survival and synthesised their overall discriminative performance. Methods: A systematic search of PubMed, Scopus, and Web of Science (2010–2024) identified studies developing ML models for 5-year survival prediction. Study quality was assessed using PROBAST. Logit-transformed area under the receiver operating characteristic curve (AUC) estimates were quantitatively synthesised using a random-effects meta-analysis with the restricted maximum likelihood (REML) estimator. Heterogeneity was assessed using Cochran’s Q and I^2^ statistics, publication bias using funnel plots and Egger’s regression test, and subgroup analyses according to study characteristics. Statistical analyses were performed using IBM SPSS version 29.0 and R version 4.6.1. Results: Fifteen studies were included. A wide range of ML models, including Random Forest, Random Survival Forest, and gradient boosting methods, was evaluated. The pooled analysis demonstrated good discriminative performance, with an overall AUC of 0.83 (95% CI: 0.80–0.86). Substantial heterogeneity was observed across studies. Ensemble-based models generally showed consistent performance. Publication bias was detected, and several studies exhibited moderate-to-high risk of bias. Conclusions: ML models show strong potential for predicting 5-year breast cancer survival and may support early risk stratification and clinical decision-making. However, greater methodological standardisation, rigorous external validation, and improved reporting are required before widespread clinical implementation.

## Simple Summary

Breast cancer is a highly heterogeneous disease, making accurate prediction of 5-year survival challenging. Machine learning has emerged as a promising approach for improving prognostic accuracy by identifying complex patterns within routinely collected clinical data. This systematic review and meta-analysis evaluated the predictive performance of machine learning models developed for 5-year breast cancer survival. Overall, the included studies demonstrated good discriminative performance, suggesting that machine learning-based models may support early identification of high-risk patients and facilitate personalised clinical decision-making. Nevertheless, methodological heterogeneity, limited external validation, and variable reporting quality remain important barriers to routine clinical implementation. Further well-designed studies with standardised reporting and rigorous external validation are required before these models can be widely adopted in clinical practice.

## 1. Introduction

Breast cancer (BC) remains one of the most common malignancies affecting women worldwide and represents a major global public health challenge. BC originates from the epithelial cells of the mammary ducts or lobules and may metastasise to distant organs if not detected and treated early [1]. The disease exhibits substantial heterogeneity in clinical presentation, biological behaviour, and therapeutic response, necessitating individualised multidisciplinary management. Despite substantial advances in diagnosis and treatment, BC continues to impose a considerable global health burden [2]. Globally, BC is the most frequently diagnosed cancer, with an estimated 2.3 million new cases and 685,000 deaths reported in 2020 [3]. According to the Global Cancer Observatory (GLOBOCAN), it accounts for approximately 11.7% of all cancer cases and 6.9% of cancer-related deaths worldwide [4].

In India, BC is the most frequently diagnosed malignancy among women, with an increasing incidence among younger populations and urban residents [5]. The incidence continues to rise globally due to increasing life expectancy, urbanisation, and the adoption of Western lifestyles. BC risk is influenced by both modifiable and non-modifiable factors. Non-modifiable risk factors include age, sex, family history, inherited genetic mutations, and reproductive characteristics such as early menarche and late menopause [6]. Modifiable risk factors include obesity, physical inactivity, alcohol consumption, hormone replacement therapy, and exposure to ionising radiation [7]. Limited access to healthcare and socioeconomic disparities contribute to delayed diagnosis and poorer outcomes, particularly in low- and middle-income countries [8].

The management of BC typically involves a multidisciplinary approach incorporating surgery, radiotherapy, chemotherapy, hormonal therapy, and targeted biological agents, tailored to the tumour stage and molecular subtype [9]. Early-stage disease is commonly managed with breast-conserving surgery followed by radiotherapy, whereas advanced disease often necessitates systemic therapy, including targeted agents such as trastuzumab for HER2-positive tumours [10]. Recent advances in precision medicine and immunotherapy have further transformed breast cancer treatment by enabling more personalized therapeutic strategies [11].

Traditionally, survival prediction in BC has relied on statistical approaches, such as Cox proportional hazards regression and staging-based prognostic systems. Although these methods have contributed significantly to clinical practice, they are constrained by assumptions of proportional hazards and linearity, as well as by limited capacity to model complex interactions among prognostic variables [12]. Because BC outcomes are influenced by nonlinear relationships among demographic, pathological, and treatment-related factors, these limitations may reduce predictive accuracy in real-world clinical settings. In recent years, ML techniques have emerged as alternatives for survival prediction in oncology. ML models can handle high-dimensional data, accommodate nonlinear effects, and capture complex interactions without prespecifying functional forms [13]. Consequently, several studies have applied ML algorithms, including Random Forest (RF), Support Vector Machines (SVMs), Neural Networks (NNs), and ensemble methods, to predict survival outcomes in BC using routinely collected clinical and pathological data [14].

Although the application of ML techniques in BC prognosis is increasing, the current body of evidence remains fragmented and methodologically heterogeneous. Existing studies report widely varying performance estimates due to differences in study design, sample size, predictor selection, algorithm choice, and validation strategies [15]. Moreover, most studies rely predominantly on internal validation approaches, such as cross-validation or random data splitting, with limited external validation in independent populations. This raises important concerns regarding overfitting, optimism bias, and generalizability of the reported model performance [16].

Despite the rapid growth of ML applications in BC prognosis, there remains a lack of quantitative synthesis assessing the overall predictive performance of ML-based models for 5-year survival outcomes. Existing reviews are predominantly narrative in nature or focus on broader oncology applications, diagnostic imaging, or molecular prediction rather than on long-term survival [17]. Notably, there is a lack of rigorous meta-analytical approaches that systematically pool model performance using standardised discrimination metrics, such as the Area Under the Receiver Operating Characteristic Curve (AUC) or Concordance Index (C-index). This gap limits the ability to draw robust and generalizable conclusions about the true predictive performance and clinical utility of ML-based models for 5-year BC survival. Accordingly, this systematic review and meta-analysis aimed to synthesise existing evidence on ML models developed using routinely collected clinical and pathological variables for predicting 5-year BC survival, pool key performance metrics, and critically evaluate their methodological quality and clinical applicability.

## 2. Materials and Methods

### 2.1. Protocol Registration

The study was conducted and reported in accordance with the Preferred Reporting Items for Systematic Reviews and Meta-Analyses (PRISMA) guidelines [18]. It was registered with the International Open-Access Prospective Register of Systematic Reviews (PROSPERO). The PROSPERO registration number is CRD420251066355.

### 2.2. Search Strategy

Two independent investigators systematically searched three databases, including PubMed, Scopus, and Web of Science, from 2010 to 2024. The search strategies employed a combination of keywords, Boolean operators, wildcards, and database-specific truncation rules. The search items included Medical Subject Headings (MeSH) and free-text terms, such as “Breast cancer”, “Machine learning”, “Survival”, and “Prediction”. Discrepancies were resolved by consensus. Details regarding the search terms for all databases are presented in Supplementary Materials, Table S1.

### 2.3. PICO Search Guide

Population (P): Female patients diagnosed with BC, irrespective of disease stage and histological subtype.

Intervention (I): Application of ML-based prediction models developed using routinely collected clinical and pathological variables to estimate 5-year BC survival outcomes.

Comparator (C): Comparisons between ML models and traditional statistical approaches were considered.

Outcome (O): The outcome of interest was model performance in predicting 5-year survival, primarily assessed using discrimination metrics such as AUC.

### 2.4. Study Outcome

The primary outcome of this study was the discriminative performance of ML-based prediction models for estimating 5-year survival in patients with BC, evaluated primarily using the AUC. Secondary performance measures, including the C-index, accuracy, sensitivity, and specificity, were extracted wherever available.

### 2.5. Inclusion and Exclusion Criteria

The inclusion criteria were as follows: (1) published peer-reviewed primary research; (2) studies on the clinical diagnosis and prognosis of female patients with BC; (3) studies that employed ML algorithms to develop models predicting 5-year BC survival; (4) reporting at least one performance metric (AUC, accuracy, sensitivity, specificity, or C-index); and (5) full-text articles published in English.

The exclusion criteria were as follows: (1) review articles, editorials, conference abstracts, or commentaries; (2) studies predicting less than 5-year survival of BC using ML models; (3) studies based on non-clinical data (imaging, genomic, or drug-related data); (4) studies not focused on female BC; (5) studies that do not clearly describe model development procedures, including data splitting, model training, and validation strategy; (6) studies lacking data on performance metrics; and (7) animal studies and published in languages other than English.

### 2.6. Data Extraction

Data extraction was independently performed by two reviewers using a structured approach to ensure completeness and consistency across all included articles. Any discrepancies were resolved through discussion. The initial literature screening was performed using Microsoft Excel 2019 and COVIDENCE. Titles and abstracts were first reviewed to eliminate irrelevant studies, followed by a full-text assessment of the remaining articles to determine their eligibility for inclusion in the present study.

The extracted information included the following components:

- Basic study information: First author, year of publication, and country of the study.
- Study characteristics: Data source, study design, study period, population characteristics (age group and specific BC subtype), and type of survival outcome (overall survival, disease-free survival, or disease-specific survival).
- ML model details: Sample size, train–test split ratio, cross-validation method, validation approach (internal or external), feature selection or extraction technique, and ML model applied.
- Model performance metrics: AUC, accuracy, sensitivity, specificity, and C-index.

### 2.7. Assessment of Risk of Bias

The risk of bias and applicability were assessed using the Prediction Model Risk of Bias Assessment Tool (PROBAST) [19] across the four key domains of participants, predictors, outcome, and analysis, applying all 20 signalling questions relevant to studies developing prediction models. Two reviewers independently evaluated the bias tool, with judgments recorded as “yes”, “probably yes”, “probably no”, “no”, or “no information”. Any discrepancies were resolved through discussion to reach a consensus. Domain-level and overall ratings were classified as low, high, or unclear risk of bias according to the PROBAST guidance. The overall study risk of bias was considered high if any domain was rated high. Applicability concerns were evaluated separately for participants, predictors, and outcomes with reference to the review question.

### 2.8. Statistical Analysis

A random-effects model was used to estimate pooled AUCs with 95% confidence intervals (CIs), reflecting the overall discriminative performance of the included ML models. Given the anticipated clinical and methodological heterogeneity across studies, including differences in patient populations, survival outcomes, predictors, validation strategies, and modelling approaches, a random-effects model was selected a priori. Although the included studies evaluated different survival outcomes, including OS, DFS, and DSS, all focused on predicting 5-year survival-related outcomes in patients with BC. Given the methodological diversity across studies, the pooled AUC estimates were intended to provide an overall summary measure of discriminative performance rather than a common effect size. Therefore, pooled estimates should be interpreted as descriptive summaries of the existing literature.

Only AUC values were included in the quantitative meta-analysis, whereas other performance measures, including the C-index, were summarised descriptively. Because AUC values are bounded between 0 and 1, all reported AUCs were transformed using the logit function prior to meta-analysis to stabilise variances and ensure that pooled confidence intervals remained within the permissible range. For studies that did not report confidence intervals or standard errors, the standard error of the AUC was estimated using the normal approximation method. The corresponding standard error on the logit scale was then obtained using the delta method. Random-effects meta-analysis was performed using the restricted maximum likelihood (REML) estimator, and pooled estimates together with their 95% confidence intervals and prediction intervals were back-transformed to the original AUC scale using the inverse-logit function for interpretation.

Heterogeneity was assessed using Cochran’s Q test and the I^2^ statistic. Publication bias was evaluated using funnel plots and Egger’s regression test. Model-specific and subgroup analyses were conducted according to outcome type, validation strategy, study characteristics, and model category to explore potential sources of heterogeneity. A two-sided *p*-value < 0.05 was considered statistically significant. A sensitivity analysis was also performed by excluding studies based on the SEER database and repeating the random-effects meta-analysis to evaluate the influence of SEER-derived studies on the pooled AUC estimates. All statistical analyses were performed using IBM SPSS Statistics (version 29.0) and the R program (version 4.6.1) to ensure robustness and reproducibility.

## 3. Results

### 3.1. Study Selection

**Figure 1:**
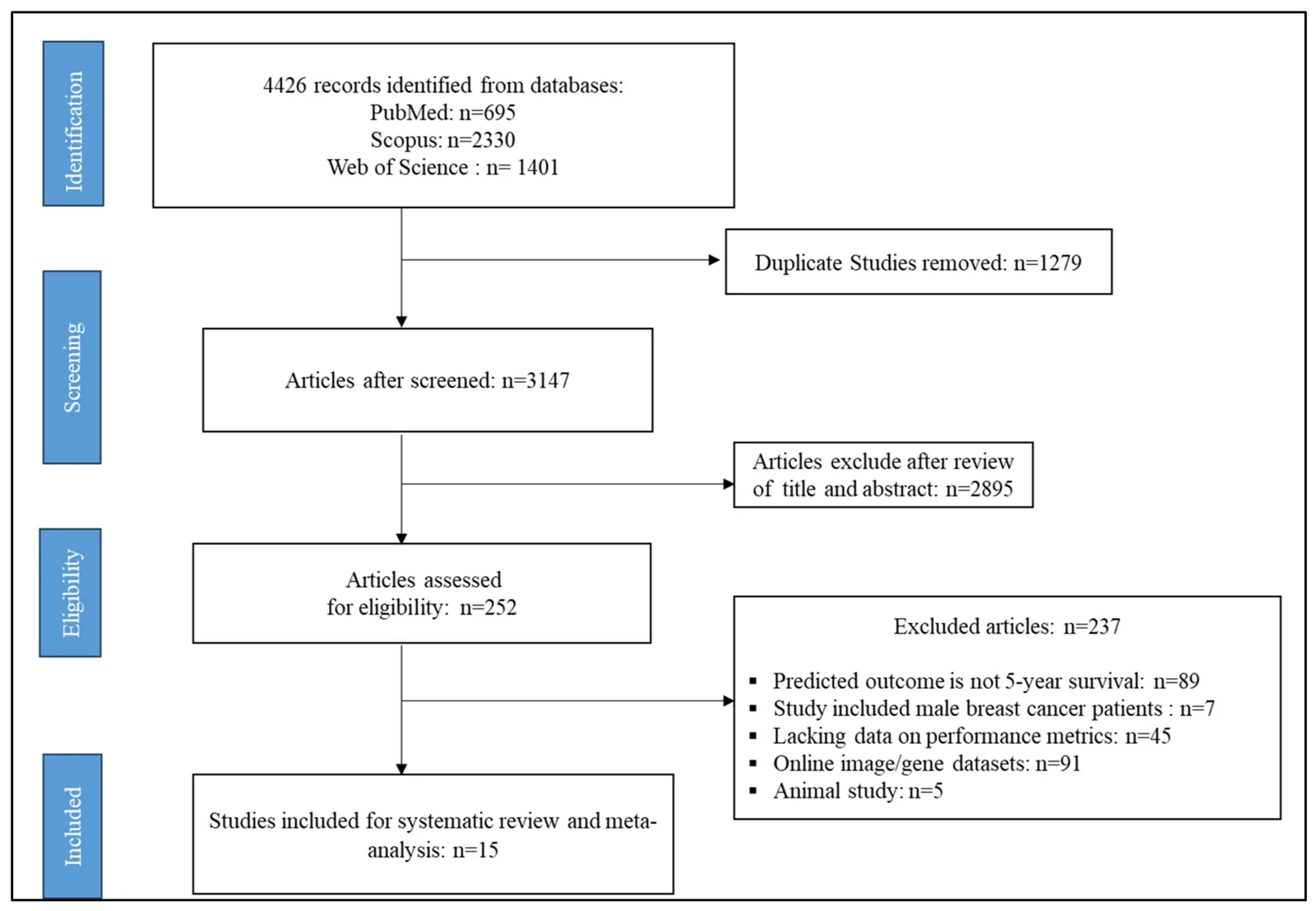
PRISMA flowchart of the study selection.

A total of 4426 records were identified from PubMed (695), Web of Science (1401), and Scopus (2330). After removing 1279 duplicate studies, 3147 studies remained. Subsequently, 2895 studies were eliminated during title and abstract screening for not meeting inclusion criteria, such as review articles, conference abstracts, book chapters, and non-English studies. Of the remaining 252 records, the full-text articles were carefully reviewed, and 237 records were excluded for the following reasons: (1) predicted outcome was not 5-year survival (*n* = 89); (2) study included male BC (*n* = 7); (3) lacking data on performance metrics (*n* = 45); (4) online image or gene datasets (*n* = 91); and (5) animal study (*n* = 5). A total of 15 studies met the inclusion criteria [20,21,22,23,24,25,26,27,28,29,30,31,32,33,34] and were included in the systematic review and meta-analysis. The study selection process followed the PRISMA guidelines and is illustrated in Figure 1.

The distribution of records by database is shown in Supplementary Figure S1, with Scopus accounting for the largest share (52%), followed by Web of Science (32%) and PubMed (16%). Figure S2 presents the types of documents from each database, with original research articles forming the majority in all three sources (Scopus 1418; Web of Science 1106; PubMed 409), reviews (Scopus 225; Web of Science 126; PubMed 68), and other publication categories (Scopus 687; Web of Science 169; PubMed 218). Figure S3 shows a clear upward trend in publications, rising from 26 in 2010 to 1197 in 2024, with sharper growth after 2018. Figure S4 shows the distribution of studies by cancer type from keyword searches, indicating that BC accounts for the largest proportion.

### 3.2. Primary Characteristics of the Literature

The systematic review comprised 15 studies published between 2020 and 2024, as shown in Table 1. Among the included studies, three were conducted in the USA [20], Iran [21], and Taiwan [28], while a further 12 originated from China [22,23,24,25,26,27,29,30,31,32,33,34]. Most studies utilised the SEER database [20,22,23,25,26,29,30,32,34], while three studies used hospital registry data [24,27,31], and one study was based on a multicenter hospital consortium [34]. Two studies combined SEER with hospital datasets [24,26], and one used the Taiwan Cancer Registry with the TMUCRD [28].

All studies adopted a retrospective cohort design [20,21,22,23,24,25,26,27,28,29,30,31,32,33,34]. The study periods ranged from 1975 to 2022, with the most common coverage between 2010 and 2019 [20,22,23,25,29,30,32]. Populations varied, including all-age BC patients [20,21,23,25,27,28,29,31,32,33], elderly patients aged ≥70 years [22], young breast cancer patients <40 years [26], early-stage invasive breast cancer [34], occult breast cancer [29], and patients with specific conditions such as invasive micropapillary carcinoma [23], bone metastases [25], and post-neoadjuvant chemotherapy [24,30]. Regarding survival outcomes, most studies reported OS [20,21,22,23,25,26,28,29,30,31,32,33], while fewer reported DFS [24,27,34] and DSS [22,30]. ML algorithms varied widely, with frequent use of RF [20,21,23,24,25,28,29,30,31,32,34], LR [20,21,24,28,29,31], SVM [21,22,23,25,28,29,30,31,32,34], and gradient boosting methods (GBoost, XGBoost, LightGBM, CatBoost) [21,22,23,25,27,28,29,30,31,32]. Some studies have also applied deep learning models, such as ANN [28] and MLP [21,31].

Model performance was primarily assessed using the AUC [20,21,22,23,24,25,26,27,28,29,30,31,32,33,34], with several studies also reporting accuracy, sensitivity, and specificity [20,21,22,24,25,28,29,31,32,34]. A few studies included the C-index [26,33] as an additional evaluation metric for the model performance.

Figure S5 shows the different ML models used in the included studies. Tree-based algorithms constituted the most frequently applied group of methods, with RF, RSF, and DT models being reported in 10, 4, and 5 studies, respectively. Boosting-based techniques were also prominently represented, including XGBoost (9 studies), GBoost (4 studies), LGBM (3 studies), and AdaBoost (3 studies). Among margin-based classifiers, SVM was employed in 9 studies. Distance-based learning methods, such as KNN, were used in 8 studies, whereas linear classification and regression approaches, particularly LR, were applied in 8 studies. Probabilistic classifiers were used moderately, with NB and MNB reported in 3 and 1 studies, respectively. Neural network-based approaches were relatively infrequent, including MLP (2 studies), ANN (1 study), and NN models (1 study). Discriminant analysis techniques, namely LDA and QDA, appeared in 2 and 1 studies, respectively. Ensemble and hybrid classifiers, such as VC and CatBoost, were rarely adopted, with each appearing in only a single study. Other specialised methods, including MARS, EXSA, and Cox-EN, were observed in only one study each.

Table 2 summarises the key characteristics and methodological features of the fifteen included studies [20,21,22,23,24,25,26,27,28,29,30,31,32,33,34]. The sample sizes varied widely, from as few as 514 patients [24] to 63,145 patients [32], reflecting both small hospital-based datasets and large national registries. Five studies included fewer than 2000 participants [21,23,24,26,29], while three studies evaluated datasets with more than 20,000 patients [20,32,33]. The most common train–test split ratios were 70:30 [25,26,27,29,30,33] and 80:20 [21,22,23,24,31]. Other approaches included 50:50 [20], 63:37 [28], and 90:10 [32,34]. Cross-validation has been widely used to improve model reliability. Ten-fold validation was the most frequently used method [20,22,23,25,26,30], followed by five-fold [21,28,31] and nine-fold [32] validation. A few studies did not mention using the cross-validation method [24,27,29,33,34]. Most studies relied on internal validation [20,22,23,25,27,28,29,30,31,32,33,34], while external validation was performed when independent datasets or multicenter cohorts were available [21,24,26]. The feature selection and extraction methods varied considerably. Common techniques included correlation analysis [20], univariate and multivariate Cox regression [22,23,25,29,34], LASSO regression [26,30], Elastic Net [33], and clinical or expert-based feature selection [21,24,28,31,32]. Some studies also used more specialised approaches, such as PSM [22], *t*-test-based filtering [34], and XGBoost-based feature optimisation [27].

LR was used in studies [20,21,22,24,28,29,31,32,34]. RF was applied in [20,21,22,23,24,28,29,31,32,34]. DT was used in [21,22,25,29,32]. ET was employed in [21,23]. GBoost was used in [21,22,27,28]. XGBoost was applied in [21,22,23,25,27,28,29,31,32]. SVM was used in [21,22,23,25,29,31,32,33,34]. KNN was employed in [21,22,23,25,29,31,32,34]. NB was applied in [21,28,32,34]. MLP was used in [21,31]. ANN was applied in [28]. AdaBoost was used in [21,28,34]. LightGBM was employed in [22,28,31]. CatBoost was used in [22]. NN was applied in [22]. RSF was used in [26,27,30,33]. Cox-EN was applied in [33]. EXSA was used in [27]. A VC was applied in [28]. LDA was used in [28,32]. QDA was applied in [32]. MARS were used in [32]. MNB was applied in [31]. The best-performing models differed between the studies. RF was identified as the top model in four studies [20,21,24,34]. XGBoost also performed best in four studies [23,25,29,31], and RSF was selected as the leading model in three studies [26,30,33]. LightGBM [22], EXSA [27], ANN [28], and MARS [32] emerged as the top performers in individual studies. AUC values ranged from 0.75 [20] to 0.96 [21], and most studies reported AUCs above 0.80 [22,23,25,27,28,29,30,31,32,33,34], indicating a strong predictive performance. The highest AUC values were achieved by RF (0.96 [21]) and ANN (0.95 [28]), whereas the lowest AUC was reported for RF (0.75 [20]).

### 3.3. Risk of Bias

Table S2 presents the risk-of-bias assessment for all included studies across the four PROBAST domains: participants, predictors, outcomes, and analysis. In the participants’ domain, all studies [20,21,22,23,24,25,26,27,28,29,30,31,32,33,34] were rated as having a low risk of bias because they included well-defined BC populations and used appropriate, representative sampling. The predictor domain also received a low-risk rating across all studies [20,21,22,23,24,25,26,27,28,29,30,31,32,33,34] because the variables were clearly described, clinically relevant, and recorded prior to outcome measurement. The outcome domain was consistently judged as low risk across studies because survival outcomes were clearly defined and objectively measured.

However, differences were observed in the analysis domain. Seven studies showed some concerns, with a moderate risk rating [22,23,24,25,33,34], mainly due to limited handling of missing data, inadequate reporting of calibration, or incomplete internal validation procedures. Four studies [26,27,29,30] were considered high risk in the analysis domain because they lacked appropriate safeguards against overfitting, did not use robust validation methods, or did not provide sufficient detail about their modeling processes. The remaining studies [20,21,28,31,32] were rated as low risk because they used appropriate validation techniques and provided clear descriptions of their analytical methods.

### 3.4. Meta-Analysis

**Figure 2:**
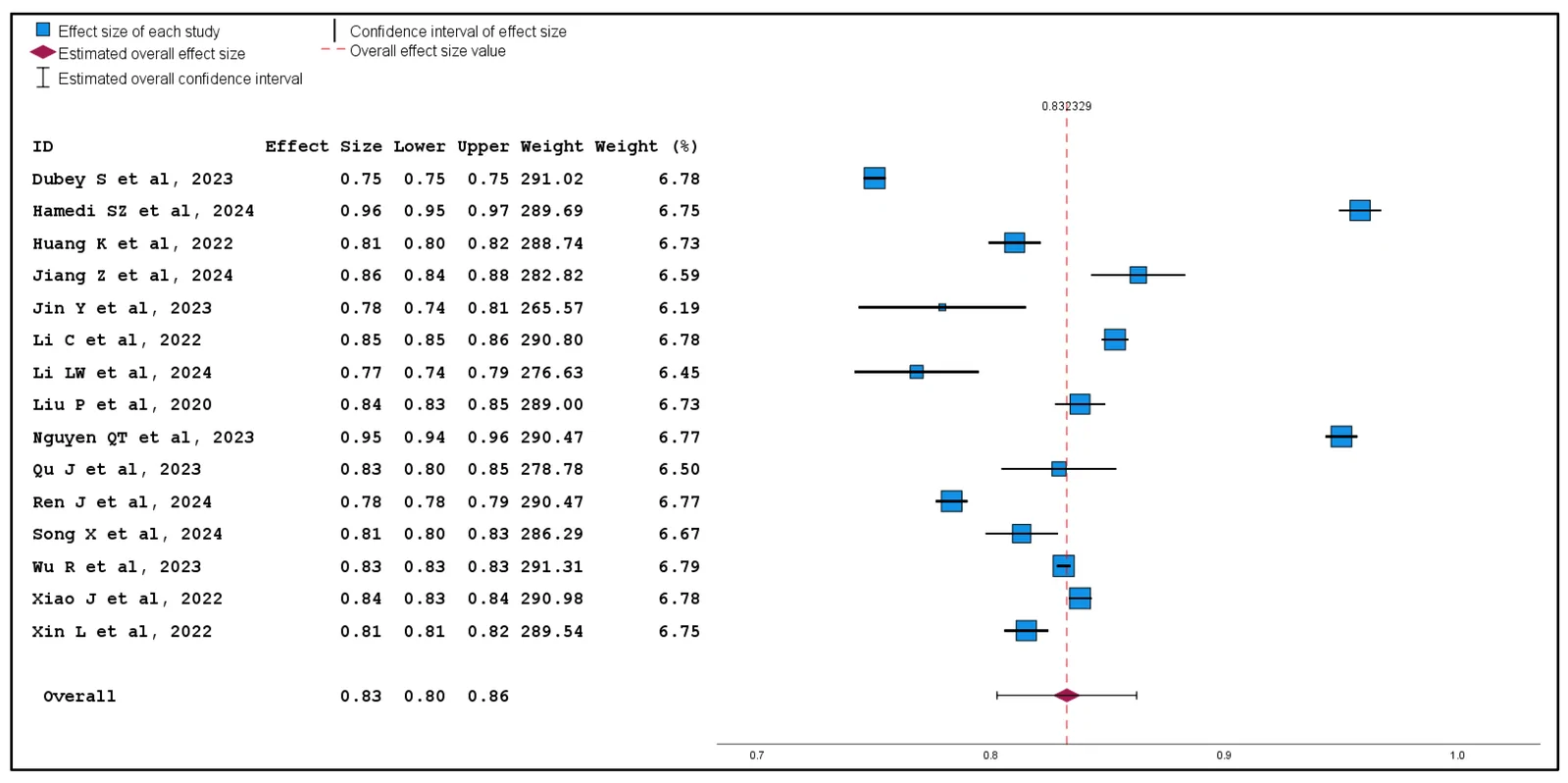
Forest plot of the best ML-based models for BC prediction [20,21,22,23,24,25,26,27,28,29,30,31,32,33,34].

**Figure 3:**
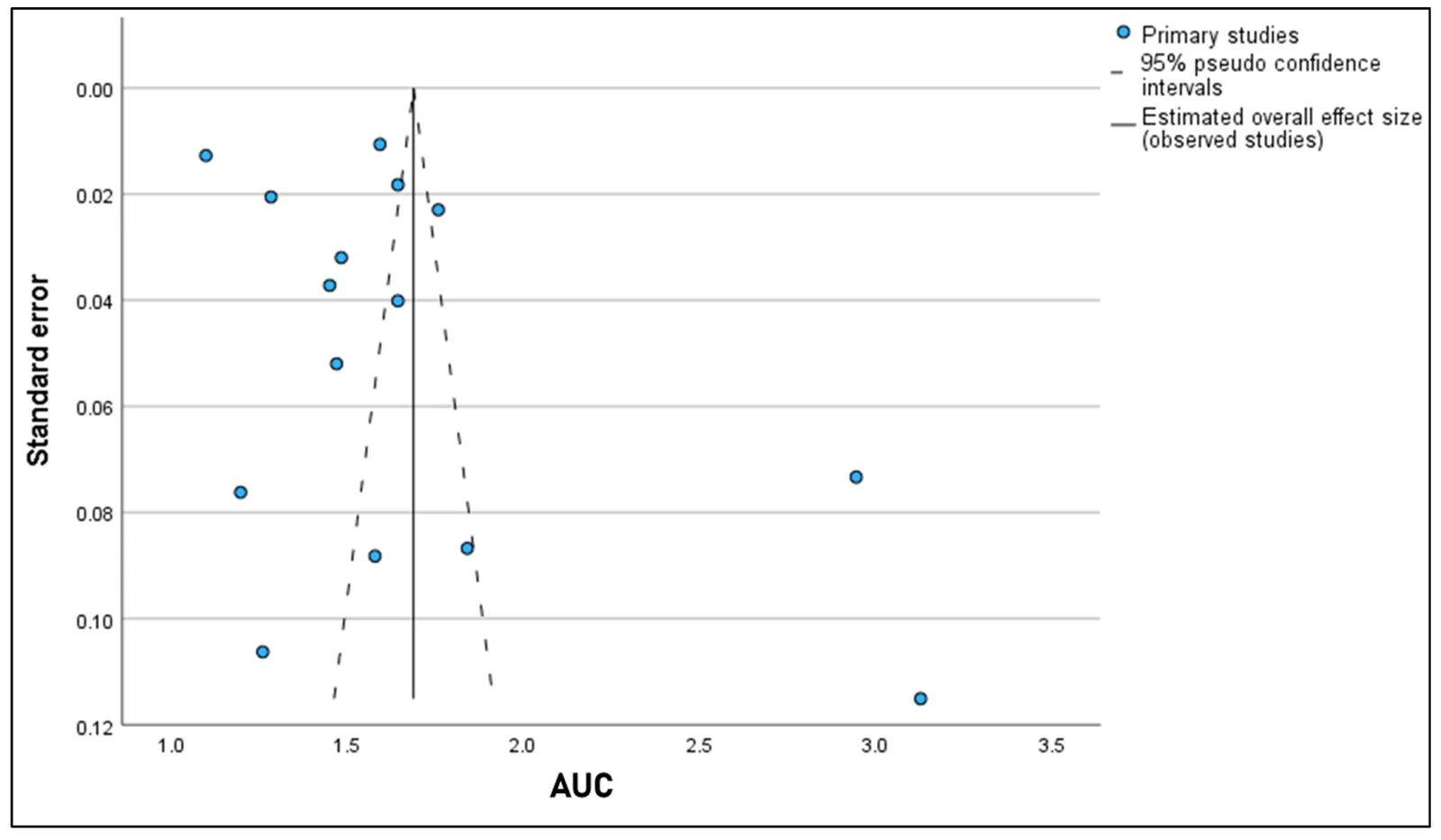
Funnel plot for assessing publication bias among the included ML-model-based studies.

The forest plot in Figure 2 presents the pooled effect estimates from the 15 studies [20,21,22,23,24,25,26,27,28,29,30,31,32,33,34]. Individual study estimates varied, ranging from 0.71 (95% CI: 0.64–0.79; [26]) to 0.96 (95% CI: 0.95–0.97; [21]), indicating considerable variability across studies. Studies such as [20,25,34] had relatively narrow CIs, indicating higher precision, whereas [24,28] had wider intervals, reflecting lower precision. The combined pooled effect was 0.83 (95% CI: 0.80–0.86), represented by the diamond at the bottom of the plot. Because the CI did not cross the no-effect line, the overall estimate was statistically significant. The narrow CI around the pooled effect also demonstrates the stability of the result, and most study estimates were close to the pooled effect line, supporting the overall consistency of the findings. However, heterogeneity testing revealed substantial inconsistency across studies, with H^2^ = 482.39, Q-statistic = 1982.69, and I^2^ = 99.8%. The very high I^2^ value indicates that nearly all the variability in the pooled AUC is due to between-study heterogeneity rather than chance. The large H^2^ and Q-statistic suggest a strong dispersion in the pooled AUCs. In terms of study weights, no single study dominated the analysis, with contributions ranging from 6.19% [24] to 6.79% [32], ensuring a balanced influence across the dataset. Overall, the meta-analysis provided evidence of a statistically significant effect; however, further subgroup analyses were conducted to explore the potential sources of variation. The funnel plot in Figure 3 shows slight asymmetry, and Egger’s regression test supported this observation, with a significant *p*-value (*p* < 0.001), suggesting a potential risk of publication bias.

### 3.5. Individual Models Meta-Analysis

The individual model-specific meta-analysis Table S3 showed generally good discriminatory performance across all ML algorithms, with pooled AUCs ranging from 0.76 to 0.89. ET (0.89; 95% CI: 0.54–0.98) and MLP (0.88; 95% CI: 0.56–0.98) had the highest pooled AUCs, followed by GBoost (0.86; 95% CI: 0.75–0.93) and XGBoost (0.84; 95% CI: 0.78–0.89), although ET and MLP estimates were imprecise due to only two studies each. RF, with the largest evidence base (n = 10), showed a pooled AUC of 0.82 (95% CI: 0.76–0.87), while Others (0.83; 95% CI: 0.77–0.87), NB (0.82; 95% CI: 0.66–0.92), LDA (0.80; 95% CI: 0.76–0.83), LGBM (0.80; 95% CI: 0.79–0.82), and RSF (0.80; 95% CI: 0.77–0.83) showed comparable performance. Simpler models such as LR (0.79; 95% CI: 0.70–0.85), DT (0.78; 95% CI: 0.68–0.85), SVM (0.77; 95% CI: 0.68–0.85), and KNN (0.76; 95% CI: 0.69–0.81) showed slightly lower but still good discrimination. AdaBoost achieved a pooled AUC of 0.83 (95% CI: 0.55–0.95) with wide uncertainty. High heterogeneity (I^2^ ≈ 100% for most models) was observed across analyses, limiting direct comparison and interpretation of model differences.

### 3.6. Subgroup Analysis

The subgroup analysis presented in Table 3 further explored heterogeneity across geographic region, publication year, sample size, study design, outcome type, and validation strategy. All subgroup categories demonstrated statistically significant Q-statistics (*p* < 0.01), indicating substantial residual heterogeneity within each subgroup. Among sample-size categories, the greatest heterogeneity was observed in studies with >10,000 participants (Q = 1300.40), followed by those with 1000–10,000 participants (Q = 549.25), whereas studies with <1000 participants showed comparatively lower heterogeneity (Q = 11.39). Geographic variation also demonstrated substantial heterogeneity, with non-China studies showing a higher Q-statistic (Q = 905.61) than China-based studies (Q = 349.48). Across publication years, heterogeneity was highest among studies published in 2023 (Q = 1338.02), followed by 2024 (Q = 283.62) and 2022 (Q = 108.02). Similarly, substantial within-group heterogeneity was observed across all study designs, with retrospective studies showing the highest Q-statistic (Q = 773.26), followed by multicenter retrospective cohort studies (Q = 474.75) and retrospective cohort studies (Q = 218.46). For outcome type, studies evaluating OS demonstrated considerably greater heterogeneity (Q = 1874.20) than those evaluating DFS (Q = 16.62). Regarding validation strategy, internally validated studies exhibited greater heterogeneity (Q = 1758.29) than externally validated studies (Q = 213.40). Overall, substantial residual heterogeneity persisted across all examined subgroups, particularly among studies evaluating OS, internally validated models, and studies published in 2023. However, these Q-statistics reflect heterogeneity within individual subgroups and should not be interpreted as evidence that these characteristics independently explain differences in heterogeneity between subgroups. Moreover, the limited number of studies in several categories may affect the stability and precision of subgroup-specific estimates; therefore, these findings should be interpreted cautiously and considered exploratory.

### 3.7. Sensitivity Analysis

To evaluate the influence of studies based on the SEER database on the primary findings, a sensitivity analysis was performed by excluding all SEER-based studies and repeating the random-effects meta-analysis using only the non-SEER studies presented in Figure S6. The pooled AUC for the non-SEER studies was 0.87 (95% CI: 0.80–0.92). Although substantial heterogeneity remained (I^2^ = 99.7%; Cochran’s Q = 550.90, *p* < 0.001), the pooled AUC was broadly comparable with that of the primary analysis (pooled AUC = 0.83). These findings suggest that the overall discriminative performance of the included ML models was not disproportionately influenced by studies derived from the SEER database, thereby supporting the robustness of the primary results.

## 4. Discussion

The prediction of long-term outcomes in BC, particularly 5-year survival, remains a major challenge in clinical oncology. Beyond serving as a standard epidemiological benchmark, 5-year survival directly influences therapeutic decision-making, including the selection and intensity of adjuvant therapy, duration of endocrine treatment, and use of targeted interventions. Therefore, improving prognostic accuracy is essential to advancing personalised cancer care, and ML has emerged as a promising approach to this end.

In the present systematic review and meta-analysis, evidence from 15 retrospective cohort studies conducted across diverse geographic regions, including China, the USA, Iran, and Taiwan, was synthesised to evaluate the performance of ML models in predicting 5-year BC survival. The pooled analysis suggested good discriminative performance, with a pooled AUC of 0.83 (95% CI: 0.80–0.86) for the best-performing models. These findings indicate that ML-based approaches may be useful for distinguishing patients with different survival outcomes and may support clinical risk stratification. Across the included studies, the most commonly selected predictors were age at diagnosis, tumour stage, histological grade, and tumour size, all of which are established prognostic determinants in BC. Age was included in nearly all models, reflecting its influence on tumour biology and patient resilience. These observations are consistent with the findings of Lu D et al. (2023), who identified age, tumour grade, and lymph node involvement as major predictors in survival modelling [35]. The consistent selection of these variables across studies suggests that the key determinants of BC survival remain relatively stable despite methodological differences.

The strong performance of ML models may be attributed to their ability to capture complex and nonlinear relationships among clinical variables without relying on restrictive statistical assumptions. Because BC outcomes are influenced by multiple interacting predictors, ML approaches may offer advantages in modelling nonlinear and interdependent relationships. Importantly, most models included in this review were developed using routinely available clinical and pathological variables rather than imaging, genomic, or molecular data, enhancing their potential applicability in low- and middle-income healthcare settings. Our findings are consistent with the systematic review by Li J et al. (2021), which reported substantial variability in the performance of ML-based survival models, with AUC values ranging from 0.50 to 0.97 [17]. Unlike previous reviews, the present study provides a quantitative synthesis of predictive performance across studies.

However, interpretation of the pooled estimates should be considered alongside the methodological quality and substantial heterogeneity of the included studies. The extremely high heterogeneity observed may reflect variations in sample composition, predictor distributions, feature engineering methods, preprocessing strategies, and validation frameworks across studies. Therefore, heterogeneity measures such as Cochran’s Q and I^2^ should be interpreted not only as indicators of poor comparability but also as reflections of variability across datasets and modelling pipelines. Furthermore, the pooled analysis included studies evaluating OS, DFS, and DSS, which represent clinically distinct prognostic outcomes. Although outcome type was explored through subgroup analyses, only three studies evaluated DFS and one study evaluated DSS, limiting the feasibility of conducting reliable outcome-specific meta-analyses. Consequently, the pooled AUC should be interpreted as a broad summary of discriminative performance across related BC prognostic tasks rather than as a common effect estimate for a single clinical endpoint. In addition, the PROBAST assessment identified several studies with moderate-to-high risk of bias, particularly in the analysis domain. Common concerns included inadequate handling of missing data, limited reporting of calibration assessment, insufficient safeguards against overfitting, and reliance on internal validation.

An additional consideration is that the present review synthesised studies evaluating different ML algorithms developed using distinct datasets and modelling strategies. Consequently, the objective of the meta-analysis was not to identify a single superior algorithm but rather to provide an overall assessment of the predictive discrimination reported across the literature. Differences in model performance may reflect variations in patient populations, predictor availability, feature engineering approaches, outcome definitions, and validation procedures rather than the intrinsic superiority of a particular ML technique. Therefore, comparisons between individual algorithms should be interpreted with caution. An important methodological consideration is that the included studies did not represent a single prognostic framework. Most studies developed binary classification models to predict 5-year survival status, whereas fewer used survival-specific approaches, such as RSF and Cox-EN, which explicitly account for censoring and time-to-event information. Consequently, the pooled estimates represented predictive discrimination across related but methodologically distinct prognostic tasks. Furthermore, discrimination metrics were variably reported across studies, and conventional AUC measures may not be fully equivalent to survival-specific metrics such as the C-index, which should be considered when interpreting the pooled findings. Although AUC was selected as the primary metric because it was the most consistently reported performance measure across studies, discrimination alone does not fully reflect clinical usefulness. Measures such as sensitivity, specificity, calibration, positive predictive value, and decision curve analysis provide complementary information on the clinical applicability of prognostic models. However, these outcomes were inconsistently reported and therefore could not be quantitatively synthesised. As a result, the present findings primarily reflect discriminative performance rather than the overall clinical utility of ML-based survival prediction models.

The comparative evaluation of ML algorithms demonstrated increasing use of ensemble and boosting techniques, including RF, GBoost, LightGBM, XGBoost, and RSF, all of which showed relatively strong predictive performance across studies. Among these, RF was one of the most frequently used models and demonstrated stable predictive performance, likely due to its robustness to complex, high-dimensional data. In comparison, traditional statistical approaches such as LR generally demonstrated lower predictive performance. However, differences between algorithms should be interpreted with caution, as variations in reported performance may reflect differences in study populations, predictor availability, feature engineering strategies, sample size, outcome definitions, and validation methods rather than the intrinsic superiority of a particular algorithm. Previous evidence from Huang Y et al. (2025) similarly suggested that ML models do not consistently outperform traditional statistical methods, particularly in settings with limited data complexity [36].

The identification of RSF as a high-performing method represents an important advancement in survival prediction because it accounts for censoring and time-to-event information, thereby providing more clinically relevant prognostic estimates than conventional binary classification models. Nevertheless, although discrimination measures such as AUC were commonly reported, discrimination alone does not ensure clinical usefulness. Calibration, which reflects the agreement between predicted and observed outcomes, was rarely evaluated, and only a limited number of studies assessed clinical utility using decision-analytic methods. Therefore, despite promising discrimination performance, current evidence remains insufficient to determine whether these models provide reliable risk estimates for routine clinical decision-making.

Recent evidence further supports the growing role of ML in BC research. Javanmard Z et al. (2025) reported high predictive accuracy across ML models, particularly deep learning approaches [37], whereas Albuquerque DAN et al. (2025) demonstrated that model performance may decline in external datasets, emphasising the importance of external validation [38]. In addition, studies by Albuquerque DAN et al. (2025) and Fu Y et al. (2024) highlighted the utility of AI in diagnostic and molecular prediction tasks, including biomarker identification and HER2 classification [38,39]. Although these studies focused on different clinical endpoints, they collectively demonstrate the broader applicability of ML approaches across BC care. Similarly, Hsu H et al. (2025) emphasised the importance of translating AI-based innovations into clinically meaningful applications [40]. These considerations are equally relevant to prognostic modelling, where robust external validation, clinical utility assessment, and integration into real-world workflows remain essential before routine clinical implementation.

Despite its strengths, this study has several limitations. All included studies were retrospective, which may introduce bias related to patient selection, data quality, and missing information. Most studies relied on internal validation, which may overestimate model performance and limit generalizability to independent populations. In addition, variations in data sources, feature selection strategies, and modelling approaches may have affected the consistency and reproducibility of findings. The analysis was also limited to models based on routinely available clinical and pathological variables, excluding imaging and molecular data. Furthermore, model performance was primarily assessed using discrimination measures such as AUC without adequate evaluation of calibration or clinical utility. An additional consideration is that the quantitative synthesis included only the best-performing ML model reported in each study. Although this approach was adopted to provide a single independent estimate per study, it may introduce optimism bias because the selected model may represent the highest reported performance rather than the performance expected in routine clinical practice. Therefore, the pooled AUC should be interpreted as representing the best reported discriminative performance across the included studies. The pooled estimates combined studies evaluating different survival outcomes, including OS, DFS, and DSS, which may have contributed substantially to heterogeneity and reduced the interpretability of a single summary estimate. Although subgroup analyses were conducted according to outcome type, the limited number of DFS and DSS studies precluded robust outcome-specific meta-analyses. Therefore, future evidence syntheses should evaluate these outcomes separately when a larger body of literature becomes available. Several studies were derived from the SEER database; therefore, partial overlap of patient populations cannot be completely excluded. Such overlap may underestimate the true sampling variance, potentially resulting in artificially narrow confidence intervals around the pooled estimates. Finally, the uneven geographic distribution of studies may limit the generalizability of the findings across diverse populations.

Future research should focus on improving the generalizability and clinical applicability of ML models for BC survival prediction. Prospective multicenter studies involving diverse populations are needed to validate model performance across different healthcare settings. Greater emphasis should be placed on external validation, calibration assessment, decision-curve analysis, and standardised reporting frameworks such as TRIPOD-AI to improve reproducibility and transparency. Integration of multimodal data, including imaging, genomic, and molecular information, may further improve predictive accuracy and risk stratification. In addition, future studies should prioritise geographically diverse datasets, reduce algorithmic bias, and improve model interpretability to facilitate the successful integration of ML-based decision support tools into routine oncology practice.

## 5. Conclusions

This study provides evidence that ML approaches may effectively support the prediction of long-term survival outcomes in patients with BC. Using routinely available clinical and pathological variables, these models offer a data-driven framework for prognostic evaluation and risk stratification. In clinical practice, such approaches may help clinicians identify high-risk patients earlier, thereby supporting more informed treatment decisions and optimised follow-up strategies. However, the substantial heterogeneity across studies, reliance on retrospective designs, and limited external validation highlight the need for standardised model development and rigorous validation in diverse populations. Future research should focus on improving model generalizability, calibration, and clinical applicability to ensure reliable implementation in real-world oncology settings.

## Figures and Tables

**Table 1:** Primary characteristics and details of the included studies.

Author	Year	Country	Data Source	Study Design	Population	Study Period	Age Group	Survival Type	ML Algorithms	Model Evaluation Metrics
Dubey S et al. [20]	2023	USA	SEER database	Retrospective study	BC patients	2010–2018	All age groups	OS	LR & RF	AUC, Accuracy, Sensitivity & Specificity
Hamedi SZ et al. [21]	2024	Iran	Breast Diseases Research Centre (Shiraz) & Cancer Research Centre (Tehran)	Multicenter retrospective cohort study	BC patients	2003–2013; 2008–2018	All age groups	OS	KNN, NB, DT, LR, RF, ET, SVM, Ada Boost, GBoost, XGBoost & MLP	AUC, Accuracy, Sensitivity & Specificity
Huang K et al. [22]	2022	China	SEER database	Retrospective cohort study	Early elderly triple-negative breast cancer patients	2010–2016	≥70 years	OS & DSS	KNN, CatBoost, DT, RF, GBoost, LightGBM, NN, SVM & XGBoost	AUC, Accuracy & Sensitivity
Jiang Z et al. [23]	2024	China	SEER database	Retrospective study	Patients diagnosed with invasive micropapillary carcinoma	1998–2019	All age groups	OS	SVM, KNN, RF, ET & XGBoost	AUC, Accuracy & Specificity
Jin Y et al. [24]	2023	China	First Affiliated Hospital of Chongqing Medical University dataset & SEER database	Retrospective study	BC patients with stable disease or progressive disease after neoadjuvant chemotherapy	2013–2019; 1975–2022	All age groups	DFS	LR & RF	AUC, Sensitivity & Specificity
Li C et al. [25]	2022	China	SEER database	Retrospective study	BC patients with initial bone metastases	2010–2019	All age groups	OS	XGBoost, SVM, DT & KNN	AUC, Accuracy, Sensitivity & Specificity
Li LW et al. [26]	2024	China	SEER database & Tianjin Medical University	Retrospective study	Young breast cancer	2010–2020; 2006–2021	<40 years	OS	RSF	AUC & C-index
Liu P et al. [27]	2020	China	West China Hospital	Retrospective study	BC patients	1989–2016	All age groups	DFS	RSF, GBM, XGBoost & EXSA	AUC
Nguyen QT et al. [28]	2023	Taiwan	TMUCRD (Taipei Medical University Clinical Research Database) & Taiwan Cancer Registry (TCR)	Multicenter retrospective cohort study	BC patients	2009–2020	All age groups	OS	LR, LDA, LGBM, GBoost, XGBoost, RF, Ada Boost, VC & ANN	AUC, Accuracy, Sensitivity & Specificity
Qu J et al. [29]	2023	China	SEER database	Retrospective cohort study	Occult breast cancer	2010–2019	All age groups	OS	LR, RF, XGBoost, DT, KNN & SVM	AUC
Ren J et al. [30]	2024	China	SEER database	Retrospective cohort study	Early BC patients with post-neoadjuvant systemic therapy	2010–2018	20–79 years	DSS	RSF	AUC
Song X et al. [31]	2024	China	Sir Run Run Shaw Hospital	Retrospective study	BC patients	August 1998–November 2021	All age groups	OS	LR, SVM, RF, XGBoost, MNB, KNN, MLP & LGBM	AUC, Accuracy, Sensitivity & Specificity
Wu R et al. [32]	2023	China	SEER database	Retrospective cohort study	BC patients	2010–2014	All age groups	OS	NB, LDA, QDA, KNN, SVM, CART, RF, MARS, LR & XGBoost	AUC, Accuracy, Sensitivity & Specificity
Xiao J et al. [33]	2022	China	Fudan University Shanghai Cancer Centre	Retrospective cohort study	BC patients	2008–2016	All age groups	OS	Cox-EN, SVM & RSF	AUC & C-index
Xin L et al. [34]	2022	China	Chinese Society of Breast Surgery	Multicenter retrospective cohort study	Early-stage invasive breast cancer patients with low-positive HER2 expression	2015–2016	20–90 years	DFS	RF, SVM, KNN, LR, NBM & AdaBoost	AUC, Sensitivity & Specificity

AdaBoost (Adaptive Boosting), ANN (Artificial Neural Network), CatBoost (Categorical Boosting), Cox-EN (Cox Elastic Net), ET (Extra Trees), EXSA (Efron-based XGBoost for Survival Analysis), DT (Decision Tree), GBoost (Gradient Boosting), KNN (K-Nearest Neighbours), LDA (Linear Discriminant Analysis), LGBM (Light Gradient Boosting Machine), LR (Logistic Regression), MARS (Multivariate Adaptive Regression Splines), MLP (Multilayer Perceptron), MNB (Multinomial Naïve Bayes), NB (Naïve Bayes), NN (Neural Network), RF (Random Forest), RSF (Random Survival Forest), QDA (Quadratic Discriminant Analysis), SVM (Support Vector Machine), XGBoost (Extreme Gradient Boosting), VC (Voting Classifier), SEER (Surveillance, Epidemiology, and End Results), OS (Overall Survival), DFS (Disease-Free Survival) and DSS (Disease-Specific Survival).

**Table 2:** Methodological characteristics and modelling strategies of the included ML models.

Author	Sample Size	Train-Test Split	Cross-Validation	Validation Method	Feature Selection/Extraction	All Models	AUC Value	Best Model	AUC Value
Dubey S et al., 2023 [20]	32,922	50–50	10-fold	Internal validation	Correlation analysis	LR	0.70	RF	0.75
						RF	0.75		
Hamedi SZ et al., 2024 [21]	1875	80–20	5-fold	External validation	Based on clinical relevance	Ada Boost	0.95	RF	0.96
						DT	0.89		
						ET	0.96		
						GBoost	0.95		
						KNN	0.89		
						LR	0.94		
						MLP	0.94		
						NB	0.91		
						RF	0.96		
						SVM	0.94		
						XGBoost	0.96		
Huang K et al., 2022 [22]	4696	80–20	10-fold	Internal validation	Univariate and multivariate Cox regression, and PSM	CatBoost	0.76	LGBM	0.81
						DT	0.69		
						GBoost	0.80		
						KNN	0.73		
						LGBM	0.81		
						NN	0.79		
						RF	0.72		
						SVM	0.70		
						XGBoost	0.79		
Jiang Z et al., 2024 [23]	1123	80–20	10-fold	Internal validation	Univariate and multivariate Cox regression	ET	0.75	XGBoost	0.86
						KNN	0.74		
						RF	0.79		
						SVM	0.81		
						XGBoost	0.86		
Jin Y et al., 2023 [24]	514	80–20	NA	External validation	Based on clinical relevance	LR	0.62	RF	0.78
						RF	0.78		
Li C et al., 2022 [25]	15,129	70–30	10-fold	Internal validation	Univariate and multivariate Cox regression	DT	0.67	XGBoost	0.85
						KNN	0.60		
						SVM	0.55		
						XGBoost	0.85		
Li LW et al., 2024 [26]	966	70–30	10-fold	External validation	LASSO regression	RSF	0.77	RSF	0.77
Liu P et al., 2020 [27]	4575	70–30	NA	Internal validation	Cox regression and XGBoost optimization	EXSA	0.84	EXSA	0.84
						GBoost	0.83		
						RSF	0.81		
						XGBoost	0.83		
Nguyen QT et al., 2023 [28]	3914	63–37	5-fold	Internal validation	Based on clinical relevance	Ada Boost	0.82	ANN	0.95
						ANN	0.95		
						GBoost	0.81		
						LDA	0.78		
						LGBM	0.81		
						LR	0.75		
						RF	0.82		
						VC	0.83		
						XGBoost	0.78		
Qu J et al., 2023 [29]	906	70–30	NA	Internal validation	Univariate and multivariate Cox regression	DT	0.79	XGBoost	0.83
						KNN	0.78		
						LR	0.82		
						RF	0.82		
						SVM	0.77		
						XGBoost	0.83		
Ren J et al., 2024 [30]	13,958	70–30	10-fold	Internal validation	LASSO regression and multivariate Cox	RSF	0.78	RSF	0.78
Song X et al., 2024 [31]	2435	80–20	5-fold	Internal validation	Based on clinical relevance	KNN	0.75	XGBoost	0.81
						LGBM	0.79		
						LR	0.79		
						MLP	0.75		
						MNB	0.76		
						RF	0.81		
						SVM	0.80		
						XGBoost	0.81		
Wu R et al., 2023 [32]	63,145	90–10	9-fold	Internal validation	Based on clinical relevance	DT	0.79	MARS	0.83
						KNN	0.76		
						LDA	0.81		
						LR	0.82		
						MARS	0.83		
						NB	0.81		
						QDA	0.78		
						RF	0.81		
						SVM	0.78		
						XGBoost	0.75		
Xiao J et al., 2022 [33]	22,176	70–30	NA	Internal validation	Elastic Net and traditional Cox regression	Cox-EN	0.82	RSF	0.84
						SVM	0.82		
						RSF	0.84		
Xin L et al., 2022 [34]	6486	90–10	NA	Internal validation	*t*-test and multivariate Cox regression	Ada Boost	0.60	RF	0.82
						KNN	0.73		
						LR	0.71		
						NB	0.69		
						RF	0.82		
						SVM	0.64		

NA—Not applicable, PSM—propensity score matching, and LASSO—least absolute shrinkage and selection operator.

**Table 3:** Subgroup analysis of heterogeneity across the study characteristics.

Variable	Number of Studies	Chi-Square (Q-Statistic)	*p*-Value
Region			
China	12	349.48	<0.001 *
Non-China	3	905.61	<0.001 *
Year			
2022	5	108.02	<0.001 *
2023	5	1338.02	<0.001 *
2024	4	283.62	<0.001 *
Sample group			
<1000	3	11.39	0.003 *
1000–10,000	7	549.25	<0.001 *
>10,000	5	1300.40	<0.001 *
Study design			
Retrospective study	7	773.26	<0.001 *
Retrospective cohort study	5	218.46	<0.001 *
Multicenter retrospective cohort study	3	474.75	<0.001 *
Outcome type			
OS	11	1874.20	<0.001 *
DFS	3	16.62	<0.001 *
Validation			
Internal validation	12	1758.29	<0.001 *
External validation	3	213.40	<0.001 *

*: Statistically significant (*p* < 0.05).

## Data Availability

No new data were created or analyzed in this study. Data sharing is not applicable to this article.

## Supplementary Materials

The following supporting information can be downloaded at: https://www.mdpi.com/article/10.3390/cancers18172736/s1, Figure S1: Distribution of studies identified from each search engine; Figure S2: Distribution of articles by its type across search engines; Figure S3: Number of studies published in each year; Figure S4: Distribution of published studies across major cancer types; Figure S5: Frequency of ML models used across the included studies; Figure S6: Forest plot of the sensitivity analysis including only the non-SEER studies [21,26,27,28,31,33,34]; Table S1: Search Strategy of databases; Table S2: Risk of bias grading using PROBAST for included prediction model studies and Table S3: Performance of Individual Models Across Included Studies.

## Abbreviations

The following abbreviations are used in this manuscript:

AUC	Area Under the Receiver Operating Characteristic Curve
BC	Breast Cancer
CI	Confidence Interval
C-index	Concordance Index
DFS	Disease-Free Survival
DSS	Disease-Specific Survival
HER2	Human Epidermal Growth Factor Receptor 2
ML	Machine Learning
OS	Overall Survival
PRISMA	Preferred Reporting Items for Systematic Reviews and Meta-Analyses
PROBAST	Prediction Model Risk of Bias Assessment Tool
PROSPERO	Prospective Register of Systematic Reviews
SEER	Surveillance, Epidemiology, and End Results
