# Supplementary material for: Evaluating the Performance of Machine Learning Models for Predicting 5-Year Breast Cancer Survival: A Systematic Review and Meta-Analysis
Source: Cancers (Basel). 2026 Aug 23;18(17):2736. doi: 10.3390/cancers18172736 (PMC13565122; doi:10.3390/cancers18172736)
Supplement: Supplementary file 1 [file cancers-18-02736-s001.zip › cancers-4417460-supplementary.pdf]

Evaluating the Performance of Machine Learning Models for Predicting 5-Year Breast Cancer Survival: A Systematic Review and Meta-Analysis

Ashin Krishna Chalil [1], Bindu Therayangalath [2] , Vikram Patil [3] and Chaithra Nagaraju [1,*]

[1] Division of Medical Statistics, School of Life Sciences, JSS Academy of Higher Education and Research, Mysuru 570015, India; 24plm050@jssuni.edu.in
[2] Department Cancer Registry & Epidemiology, Malabar Cancer Centre, Postgraduate Institute of Oncology Science & Research, Kannur 670103, India; bindut@mcc.kerala.gov.in
[3] Department of Radio Diagnosis, JSS Medical College & Hospital, JSS Academy of Higher Education and Research, Mysuru 570015, India; vikrampatil@jssuni.edu.in
* Correspondence: chaithra.mstats@jssuni.edu.in

Supplementary Table S1: Search strategy of databases

Search Strategy		
**Database**	**Search Query**	
**Scopus**	#1	TITLE-ABS-KEY ("breast cancer" OR "breast carcinoma" OR "breast neoplasm" OR "breast malignancy" OR "ductal carcinoma" OR "lobular carcinoma")
	#2	TITLE-ABS-KEY (surviv* OR "survival analysis" OR "survival prediction")
	#3	TITLE-ABS-KEY (predict* OR prognos* OR detect* OR decid* OR decision* OR classif*)
	#4	TITLE-ABS-KEY ("machine learning" OR "artificial intelligence" OR "deep learning")
	#5	#1 AND #2 AND #3 AND #4 AND PUBYEAR > 2009 AND PUBYEAR < 2025 AND (LIMIT-TO (LANGUAGE , "English"))
**Web of Science**	#1	TS=("breast cancer" OR "breast carcinoma" OR "breast neoplasm" OR "breast malignancy" OR "ductal carcinoma" OR "lobular carcinoma")
	#2	TS=(surviv* OR "survival analysis" OR "survival prediction")
	#3	TS=(predict* OR prognos* OR detect* OR decid* OR decision* OR classif*)
	#4	TS=("machine learning" OR "artificial intelligence" OR "deep learning")
	#5	#1 AND #2 AND #3 AND #4 AND PY=(2010-2024) AND LA=(English)
**PubMed**	#1	("breast cancer"[Title/Abstract] OR "breast carcinoma"[Title/Abstract] OR "breast neoplasm"[Title/Abstract] OR "breast malignancy"[Title/Abstract] OR "ductal carcinoma"[Title/Abstract] OR "lobular carcinoma"[Title/Abstract])
	#2	("surviv*"[Title/Abstract] OR "survival analysis"[Title/Abstract] OR "survival prediction"[Title/Abstract])
	#3	("predict*"[Title/Abstract] OR "prognos*"[Title/Abstract] OR "detect*"[Title/Abstract] OR "decid*"[Title/Abstract] OR "decision*"[Title/Abstract] OR "classif*"[Title/Abstract])
	#4	("machine learning"[Title/Abstract] OR "artificial intelligence"[Title/Abstract] OR "deep learning"[Title/Abstract])
	#5	#1 AND #2 AND #3 AND #4 AND (2010:2024[pdat]) AND (english[Language])

The asterisk (*) is a wildcard character used to represent any sequence of characters in the search query.

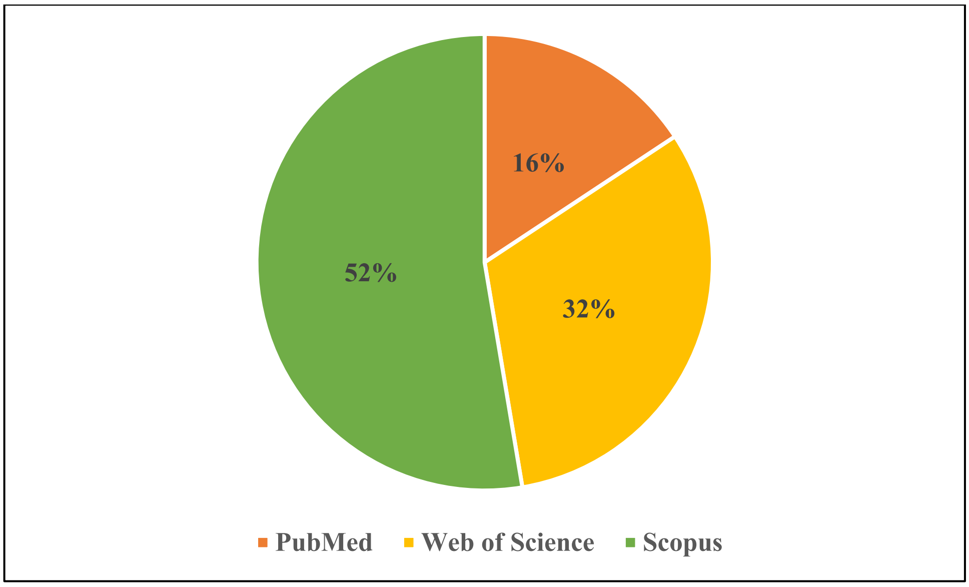

Supplementary Figure S1: Distribution of studies identified from each search engine.

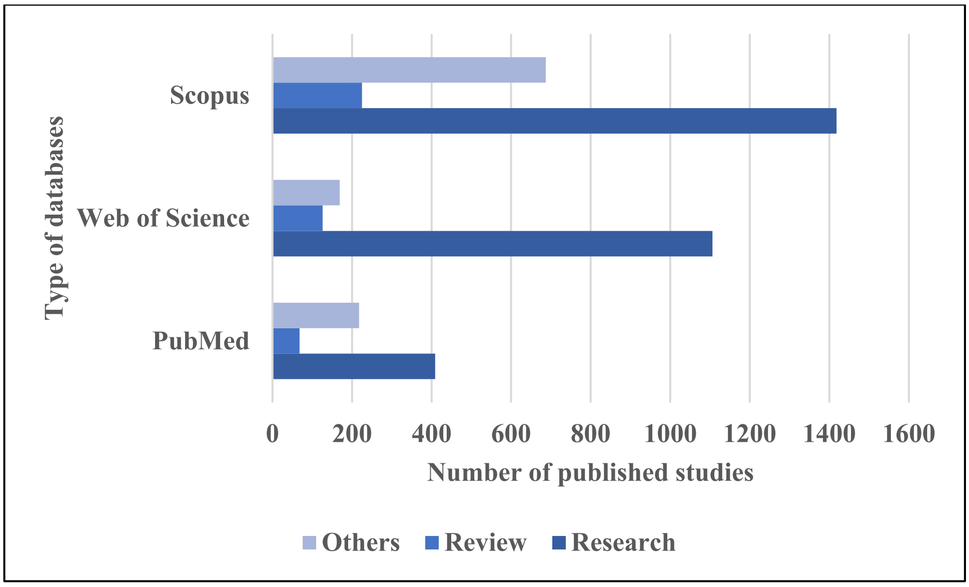

Supplementary Figure S2: Distribution of articles by its type across search engines.

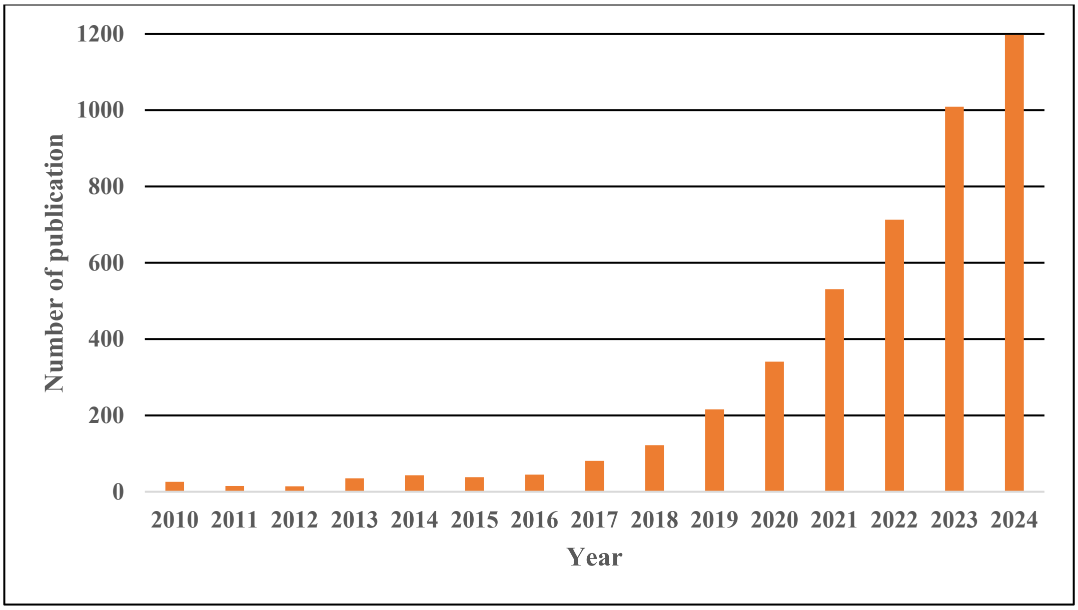

Supplementary Figure S3: Number of studies published in each year.

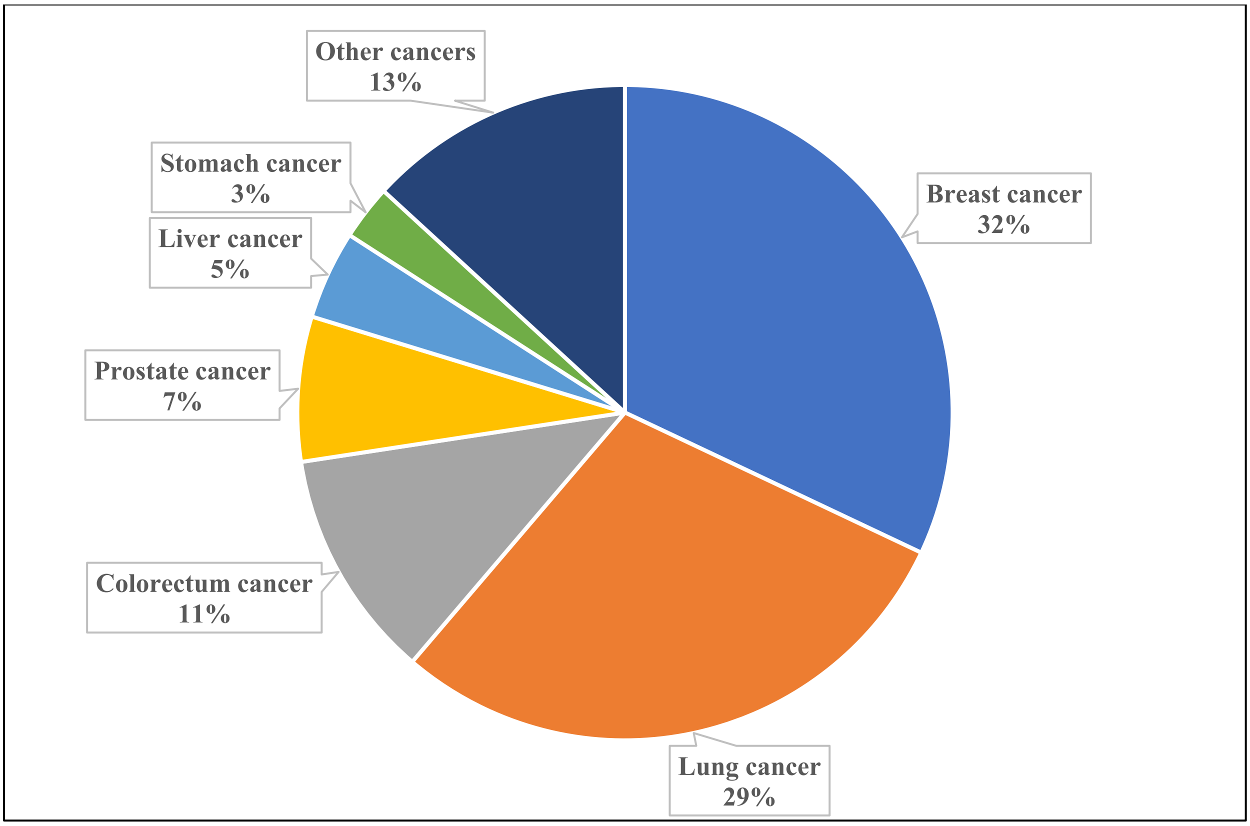

Supplementary Figure S4: Distribution of published studies across major cancer types.

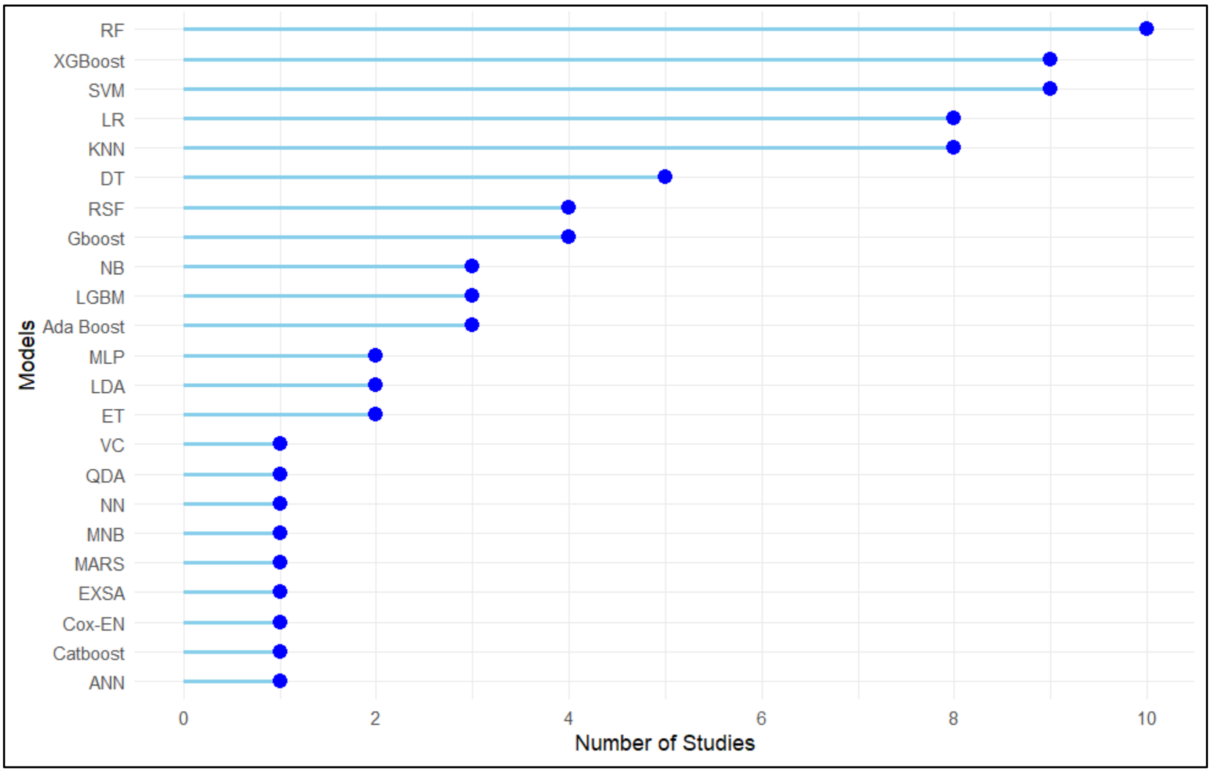

Supplementary Figure S5: Frequency of ML models used across the included studies.

Supplementary Table S2: Risk of bias grading using PROBAST for included prediction model studies.

Author	Participants bias	Predictors bias	Outcome bias	Analysis bias	Overall Risk of Bias
Dubey S et al., 2023 [20]	Low	Low	Low	Low	Low
Hamedi SZ et al., 2024 [21]	Low	Low	Low	Low	Low
Huang K et al., 2022 [22]	Low	Low	Low	Moderate	Moderate
Jiang Z et al., 2024 [23]	Low	Low	Low	Moderate	Moderate
Jin Y et al., 2023 [24]	Low	Low	Low	Moderate	Moderate
Li C et al., 2022 [25]	Low	Low	Low	Moderate	Moderate
Li LW et al., 2024 [26]	Low	Low	Low	High	High
Liu P et al., 2020 [27]	Low	Low	Low	High	High
Nguyen QT et al., 2023 [28]	Low	Low	Low	Low	Low
Qu J et al., 2023 [29]	Low	Low	Low	High	High
Ren J et al., 2024 [30]	Low	Low	Low	High	High
Song X et al., 2024 [31]	Low	Low	Low	Low	Low
Wu R et al., 2023 [32]	Low	Low	Low	Low	Low

Xiao J et al., 2022 [33]	Low	Low	Low	Moderate	Moderate
Xin L et al., 2022 [34]	Low	Low	Low	Moderate	Moderate

Supplementary Table S3: Performance of Individual Models Across Included Studies.

Models	Number of studies	I-squared(%)	Pooled AUC	95% CI	
				Lower	Upper
AdaBoost	3	100	0.83	0.55	0.95
DT	5	100	0.78	0.68	0.85
ET	2	100	0.89	0.54	0.98
Gboost	4	100	0.86	0.75	0.93
KNN	8	100	0.76	0.69	0.81
LDA	2	99.9	0.80	0.76	0.83
LGBM	3	99.4	0.80	0.79	0.82
LR	8	100	0.79	0.70	0.85
MLP	2	100	0.88	0.56	0.98
NB	3	100	0.82	0.66	0.92
RF	10	100	0.82	0.76	0.87
RSF	4	100	0.80	0.77	0.83
SVM	9	100	0.77	0.68	0.85
XGBoost	9	100	0.84	0.78	0.89
Others	9	100	0.83	0.77	0.87

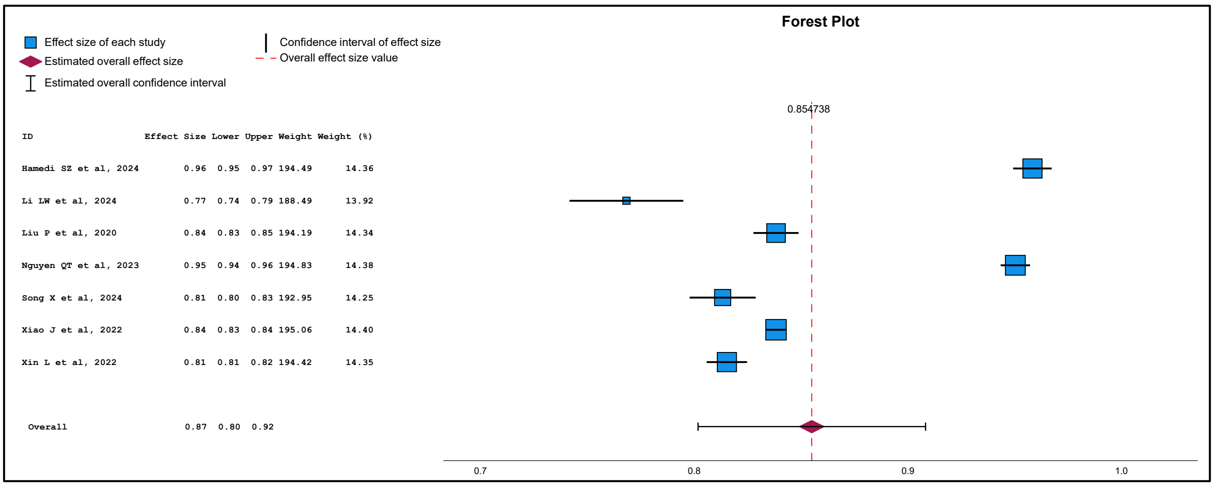

Supplementary Figure S6: Forest plot of the sensitivity analysis including only the non-SEER studies [21, 26, 27, 28, 31, 33, 34].
