# Supplementary material for: Evaluating the Performance of Machine Learning Models for Predicting 5-Year Breast Cancer Survival: A Systematic Review and Meta-Analysis
Source: Cancers (Basel). 2026 Aug 23;18(17):2736. doi: 10.3390/cancers18172736 (PMC13565122; doi:10.3390/cancers18172736)
Supplement: Supplementary file 1 [file cancers-18-02736-s001.zip › R Script for Meta.pdf]

```
############################################################
# R Script for Meta-analysis of Area Under the Curve (AUC)
# Random-effects meta-analysis using logit-transformed AUCs
############################################################

# Install packages (if required)
install.packages(c("metafor", "readxl"))

# Load packages
library(readxl)
library(metafor)

############################################################
# Import dataset
############################################################

data <- read_excel("Your_Dataset.xlsx")

############################################################
# Required variables
############################################################

# AUC          : Reported area under the ROC curve
# Sample_Size  : Total sample size of each study
# Author       : Study identifier

############################################################
# Calculate Standard Error (if not reported)
############################################################

data$SE_AUC <- sqrt((data$AUC * (1 - data$AUC)) /
```

```
                    data$Sample_Size)

############################################################
# Logit transformation
############################################################

data$Logit_AUC <- log(data$AUC /
                     (1 - data$AUC))

############################################################
# Standard error on logit scale (Delta method)
############################################################

data$SE_Logit <-
  data$SE_AUC /
  (data$AUC * (1 - data$AUC))

############################################################
# Random-effects meta-analysis
############################################################

model <- rma(
  yi = Logit_AUC,
  sei = SE_Logit,
  data = data,
  method = "REML"
)

summary(model)

############################################################
```

```
# Back-transformed pooled estimate
############################################################

predict(
  model,
  transf = plogis
)

############################################################
# Forest plot
############################################################

forest(
  model,
  slab = data$Author,
  transf = plogis,
  xlab = "Area Under the ROC Curve (AUC)",
  mlab = "Random-effects model"
)

############################################################
# Funnel plot
############################################################

funnel(
  model,
  yaxis = "sei",
  xlab = "Logit AUC"
)

############################################################
```

```
# Egger's regression test
##############################################################

regtest(
  model,
  model = "rma",
  predictor = "sei"
)

# Example subgroup analysis
##############################################################

subgroup_model <- rma(
  yi = Logit_AUC,
  sei = SE_Logit,
  data = subset(data, Group == "Example"),
  method = "REML"
)

predict(
  subgroup_model,
  transf = plogis
)

##############################################################
# Example sensitivity analysis
##############################################################

sensitivity_model <- rma(
  yi = Logit_AUC,
  sei = SE_Logit,
```

```
  data = subset(data, Source == "Non-SEER"),
  method = "REML"
)

predict(
  sensitivity_model,
  transf = plogis
)

###############################################################
# End of script
###############################################################
```
